# Note on Hydrogen Dioxide as Furnished to Practising Dentists

**Published:** 1895-02

**Authors:** Henry Leffmann


### NOTE ON HYDROGEN DIOXIDE AS FURNISHED TO PRACTISING DENTISTS.[1]

BY HENRY LEFFMANN, M.D., D.D.S.[2]

HYDROGEN DIOXIDE was for a long while a chemical curiosity, known only to a few special workers. I remember how, as a beginner in chemistry, I read with interest in one of the larger manuals the descriptions of the ingenious but tedious process by which Thénard prepared the substance, and also how much interest was awakened by its curious and exceptional properties.

Although we are still but little familiar with the pure substance, the dilute solution is now so well known that we are able to study all its important properties and utilize its valuable actions.

For several years I have kept a desultory analytical watch on the commercial solutions of hydrogen dioxide. I published in the *Medical News* over two years ago a short paper in association with Dr. William Beam, giving assays of the samples of the brands commonly sold. The examination was suggested to me by the editor of the *News*, and grew out of a paper by Dr. Wallian. In the examination a process of assay was used which had been devised by Mr. Marchand, generally accepted, and considered reliable. Dr. Squibb,

---

[1] Read before the Odontological Society of Pennsylvania, November 10, 1894.

[2] Professor of Chemistry and Metallurgy in the Pennsylvania College of Dental Surgery.

however, who at one time used and advised the method, has since shown that it exaggerates the amount of available oxygen. Nearly two years after this paper was published, one of the firms from which samples had been obtained wrote me a long letter objecting to the results. I thought that it would be a good time to reinvestigate the matter, and accordingly published in the *Medical News* the results of a much more extended series of assay, covering twenty-one samples and including various tests.

The members of this Society are, I think, mostly familiar with this paper, and I need not refer to it further than to say that it showed that while there are several excellent brands in the market, there are also many poor ones. The reputation of a house is no guarantee, nor is the price. Two samples from Merck's contained almost no dioxide. A sample which claimed as a special merit the small amount of fixed solids showed the highest in the list. Recently my attention has been called to an English brand much in favor with some physicians. I obtained a fresh sample (four-ounce bottle, retailing at one dollar), and found it to be of poor quality.

At the time the investigation of the twenty-one samples was made I had several prescriptions calling for one ounce of hydrogen dioxide put up in different parts of the city, and I also obtained samples from several of the dental depots. I was so struck by the inferiority of the latter set that I thought it well to bring the matter before this Society, but owing to my not communicating my desire to the Secretary soon enough, it could not be placed on the programme until after the article had appeared. Although a mere mention of the results in the direction was made in the *News*, I preferred to wait until a later period before reading a paper. When, therefore, I was asked by Dr. Faught to contribute something for this meeting, I suggested this topic. I have added a few facts this week, which show that there is still room for improvement.

Hydrogen dioxide solution should be a clear fluid, containing sufficient amount of the dioxide to give ten volumes of oxygen when completely decomposed. Fifty cubic centimetres of it should not require more than about five cubic centimetres of decinormal sodium hydroxide to neutralize the acid present. It should keep well in a moderately cool atmosphere. On opening a fresh sample no distinct explosion should occur, and when poured into a beaker very little effervescence should be noted.

The assay for volume strength is made by means of a solution of potassium permanganate. I need not describe it here.

I give the following figures as to hydrogen dioxide solution sold to dentists.

TESTS ABOUT FEBRUARY 16, 1894.

| | Volumes. |
|---|---|
| Prescription from A. Robbins's drug-store, Eleventh and Race Streets | 10.9 |
| Prescription from drug-store at Frankford and Girard Avenues | 10.9 |
| One-ounce bottle, S. S. White Dental Manufacturing Company | 5.1 |
| One-ounce bottle, S. S. White Dental Manufacturing Company, second sample | 5.4 |
| One-ounce bottle, G. Sibley | 0.5 |
| One-ounce bottle, H. D. Justi | 1.4 |

RECENT TESTS.

| | Volumes. |
|---|---|
| One-ounce bottle, S. S. White Dental Company, first sample, November 10 | 5.04 |
| One-ounce bottle, S. S. White Dental Company, second sample, October 30 | 5.3 |
| One-ounce bottle, H. D. Justi, first sample, November 10 | .56 |
| One-ounce bottle, H. D. Justi, second sample, November 10 | .56 |
| Sample of Marchand's purchased at G. Sibley's, October 30 | 12.04 |
| Samples of McKesson & Robbins's hydrozone, at least six months old, kept in photographic dark room, and opened to-day, November 10, no internal pressure, contains | 9.1 |
| Samples of McKesson & Robbins's pyrozone that has been standing in laboratory for at least six months, opened occasionally, showed | 9.1 |
| Pyrozone, when fresh, contains almost exactly | 10. |

I have tested also the acidity of the small samples noted above. They show a rather high acidity in some cases, but as the quantity available for test was small, it is hardly just to institute comparisons.

The above samples were all in original packages, and were tested when fresh. The one-ounce bottles cost fifteen cents,—a very high price even for a good article. Pyrozone, which is most expensive, ten-volume solution, in this market sells for fifty cents for four ounces, but the above samples are sold at the rate of sixty cents for four ounces. A ten-volume solution of excellent keeping quality is made by the Oakland Chemical Company, and may be obtained at retail at about sixty cents a pint. Such a solution will keep in an office for weeks without appreciable loss if a little care be taken not to place it in a warm corner.

There is no necessity for buying the material in one-ounce bottles, and there is also no reason that the large supply-houses should retail such inferior samples as above noted. Approximate assays are easily made at such establishments. A solution of potassium permanganate will last a long while, and but one cubic centimetre of a given sample of hydrogen dioxide is required for the test. Even, however, if this be thought too much trouble for the dealer, he can purchase guaranteed articles and retail them.

